# Laser-assisted Hair Regrowth: Fractional Laser Modalities for the Treatment of Androgenic Alopecia

**DOI:** 10.1097/GOX.0000000000002157

**Published:** 2019-04-11

**Authors:** Robert J. Dabek, William G. Austen, Branko Bojovic

**Affiliations:** From the Division of Plastic and Reconstructive Surgery, Department of Surgery, Massachusetts General Hospital, Boston, Mass.

## Abstract

**Background:**

A large proportion of the population is at sometime affected by androgenic alopecia. Current therapies consisting of minoxidil or finasteride are often the first choices for treatment. These regimens are limited by their efficacy, side-effect profiles, and often lengthy treatment courses. Low level laser/light has shown to be relatively effective and safe for the treatment of hair loss, and a number of products are currently available to consumers. Recently, fractional lasers have been examined as treatment options for androgenic alopecia. The mechanism of action of these minimally invasive resurfacing procedures is thought to be 2-fold. First, the microscopic injuries created by these treatments may induce a favorable wound healing environment that triggers hair growth. Alternatively, disruption of the stratum corneum allows for improved transdermal passage of well-established therapeutic drugs to the hair roots.

**Methods:**

A literature review was performed to evaluate the efficacy of these emerging treatments on hair regrowth.

**Results:**

Nine original studies examining the effect of fractional lasers on hair growth in androgenic alopecia have been reviewed.

**Conclusions:**

Preliminary evidence suggests that fractional laser therapies have a positive effect on hair regrowth; however, most of the literature is limited to case reports, and small prospective and retrospective series. Further studies, in the form of well-designed randomized controlled trials, are necessary to evaluate the efficacy, safety, and optimal treatment courses.

## INTRODUCTION

### Hair

Hair is an accessory appendage of the integument, which is one of the defining characteristics of vertebrates within the class Mammalia. It is composed of 2 distinct structures: the hair follicle (HF) and the shaft. HFs are situated within the dermis and contain stem cells which are responsible for hair regrowth and cutaneous wound healing. Human hairs regenerate in continuous cycles consisting of 3 stages: (1) anagen, (2) catagen, and (3) telogen. Follicle morphogenesis requires an intricately controlled regulation of apoptotic, proliferative, and differentiative signals. This regulation is often described in the literature as a “Yin and Yang” harmony.^1^ The anagen, or growth phase, is marked by the proliferation of the stem cells within the follicle to produce growth of the hair shaft. The catagen phase is manifested by involution of the HF and detachment from the underlying dermal papilla. Telogen, or resting phase, follows anagen and catagen and is marked by quiescence of the follicle. At any given time, the human scalp has hairs present within each stage of the cycle. There are a variety of cytokines and growth factors which play a role in the regulation of hair morphogenesis and transition between phases within the cycle (Table 1).^1^ However, studies involving hair growth have often demonstrated opposing conclusions regarding the role of individual growth factors on regulation of the hair cycle.^2^

**Table 1.**
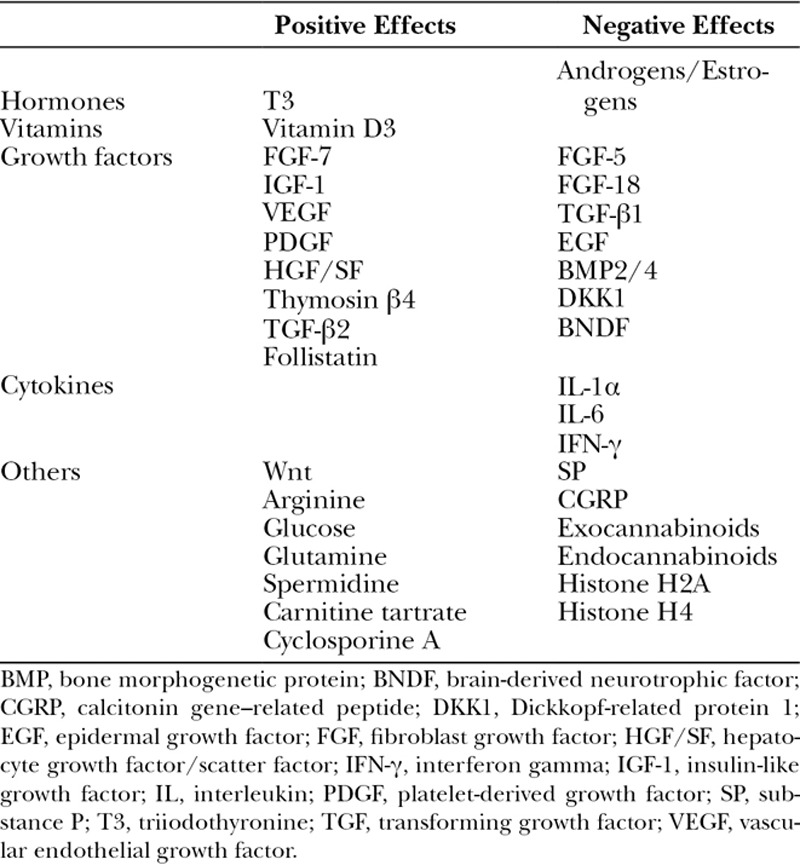
Summary of Factors Having Positive and Negative Effects on Hair Growth and Cycling

Alopecia may result when there is a disturbance of these regulatory processes, causing a reduction of hair density or a reduction of hair shaft diameter. At this time, a disproportionately higher percentage of HFs on the scalp are in the telogen phase. The cause can be attributed to 3 factors: (1) early termination of anagen, (2) prolonged telogen, or (3) failure to return to anagen. There are several disease processes that can shift the regulatory mechanisms in favor of alopecia. The most frequently studied, and most common cause is androgenic alopecia (AGA).

### Androgenic Alopecia

AGA, also known as pattern hair loss, is the most common type of alopecia, which causes loss of hair and thinning in certain areas of the scalp. Approximately 50% of women and up to 96% of Caucasian men will be affected over the course of their lives.^3,4^ The frequency and severity of AGA increases with age for both genders, but may occur as early as puberty for males. There is often a strong genetic predisposition, with autosomal dominant inheritance and incomplete penetrance.^5^ Although AGA is considered benign, psychological distress may result.^6^

AGA is considered nonscarring as there is no permanent destruction of the HF. The follicles are thought to undergo miniaturization with quiescence of the stem cells, caused by androgen hyperactivity. This relationship is not as well established in women. The alopecia is attributed to a gradual increase in transition time from telogen phase to anagen phase, and a decrease in the duration of the anagen phase.^7^ Clinically, there is patterned hair loss characterized by bitemporal recession and vertex balding in men, and diffuse loss and thinning of the crown with no loss of the temporal and frontal hairline in women.

### Treatment Options

Topical minoxidil is 1 of the 2 medical treatment measures currently approved for use in the United States by the US Food and Drug Administration (FDA). Formulation of 5% and 2% Minoxidil are available for use in both men and women. Minoxidil was originally developed as an antihypertensive medication that acts on the endothelial smooth muscle cells. Although the exact mechanism of action on hair regrowth is unknown, it likely stems from increased perfusion of the HFs leading to a cessation of follicle miniaturization. In turn, there is an increase in duration of anagen phase, and a decrease in transition time back to anagen. There are numerous randomized controlled trials supporting the use of topical minoxidil solutions for both males and females.^8,9^ Generally, daily treatment for 12 weeks is needed before substantial results are seen, and once treatment is stopped hair loss will resume within 4–6 months.^9,10^ Although complications are extremely rare, skin irritation, orthostatic hypotension, and erectile dysfunction may occur.^11^ The second FDA-approved treatment for AGA is oral finasteride. Finasteride functions as an antiandrogen, inhibiting 5α-reductase and hence reducing production of dihydrotestosterone. Many randomized controlled trials involving almost 4,000 male patients have shown that finasteride administration produces an increase in terminal hair density, compared with placebo.^8^ The hair on the male scalp in the bitemporal and vertex regions is particularly sensitive to high levels of circulating androgens and thus responds well to oral finasteride.^8^ As with topical minoxidil, the effects of treatment are not seen immediately and are transient, typically requiring about 6 months and diminishing when the medication is discontinued.^12^ The use of finasteride for women with AGA is not recommended due to unproven efficacy and concern for side effects.^13^ The side effects of oral finasteride are often a consideration when prescribing this treatment course. Most commonly sexual dysfunction can occur with a recent meta-analysis listing the relative risk as 1.39 as compared with untreated controls.^8^ Hepatic dysfunction, decreased PSA at prostate screening, and concern over fetal development in pregnant females are some of the other risks.^14^

Other treatment options for AGA with variable results include oral dutasteride, low level laser/light, ketoconazole, prostaglandin analogs, platelet-rich plasma (PRP), and a variety of surgical transplant procedures. Due to the relatively high cost and associated risks of surgical hair transplantation, the procedure is reserved for individuals who failed to respond to or cannot tolerate the use of topical minoxidil and/or oral finasteride. Hair transplants rely on the extraction of follicular units either by harvesting with a strip or small punch, and more recently with the introduction of a robotic follicular unit extractor.^15^ Results are typically excellent in appropriately selected patients.

Recently, fractional lasers have become used in the treatment of AGA and in alopecia of other causes. In general, lasers cause microtrauma or destruction of the outermost layers of the skin triggering a wound healing response.^16^ The goal of cutaneous treatments is to recruit the bodies’ mechanisms of cell growth and to regenerate the skin in a favorable fashion. This technology has been employed by plastic surgeons to treat scars, rhytids, and dyspigmentation. The increasing frequency of laser use to treat skin abnormalities, and the advent of laser hair removal saw a huge rise in the number of laser procedures being performed worldwide. Several reports began to surface of paradoxical induction of hair growth after laser hair removal procedures.^17,18^ At this time, low level laser/light technology was emerging as a treatment for alopecia.^19^ This triggered interest in exploring laser technology as a potential treatment modality. Due to the role of the HF in epidermal healing, it was thought that a stimulation of the wound healing process could in turn activate the HF. This hypothesis leads to the examination of laser-assisted hair regrowth (LAHR). In addition to the HFs’ role in epidermal wound healing, the benefits of transdermal drug delivery further substantiated the investigation of these procedures as possible treatment options. Taking advantage of increased transdermal drug delivery has become a topic of great interest and may play a considerable role in hair growth.

## METHODS

An extensive literature search was performed in June 2018 to identify publications involving fractional laser use for the treatment of AGA. PubMed/MEDLINE was searched with the following term: “fractional laser” or “fractionated laser” AND “alopecia”. Eighteen results were returned and abstracts were read to determine relevance. Seven original studies were identified as being relevant. References were reviewed and yielded an additional 2 studies. Inclusion criteria were: patient descriptions, laser therapy type and setting, and results. A similar search using Google was conducted but failed to reveal any addition publications. A total of 9 studies including prospective trials, retrospective reviews, case series, and case reports were included for final review by the authors.

## RESULTS

A total of 9 fractional laser studies, consisting of 3 mouse studies, 4 human studies, and 2 mixed human and mouse studies were reviewed (Table 2).^20–28^ One hundred and six patients, both males (n = 73) and females (n = 33), with AGA were studied. Mouse studies (n = 276 mice) used shaved mice to model the telogen phase of the hair cycle. The analysis will be organized by laser type.

**Table 2.**
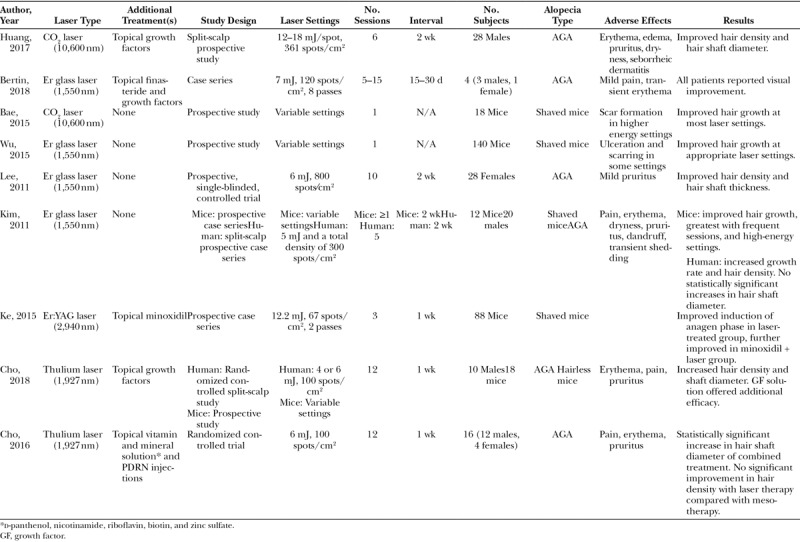
Summary of Fractional Laser Studies for AGA

### Fractional Lasers

Laser devices can be broken down into 2 categories: ablative and nonablative. Ablative laser devices cause destruction to the surface of the skin to a variable depth, vaporizing the tissue. Nonablative lasers cause damage to a lesser extent, preserving the skin (epidermal) surface. There are a number of lasers that fall into each of these categories, each with a specific wavelength and acting on a specific chromophore (target). The first laser devices acted on the entire treated surface, often resulting in a lengthy reepithelization process. The recovery time warranted a longer interval between sessions, which in turn extended the treatment course. From this, fractional lasers were developed, which acted on only a fraction of the surface.^16^ This resulted in fewer complications and allowed for less downtime and more frequent procedures, subsequently leading to more rapid results. Within our review, 4 different types of laser have been identified as being studied for the treatment of AGA.

### Fractional Ablative CO_2_ Laser

Of 2 ablative fractional laser devices, the CO_2_ laser was most commonly (n = 2) examined as a treatment for alopecia. The laser operates at a wavelength of 10,600 nm and effectively targets water, making it an excellent choice for ablation of the skin surface. The potential use of a fractional CO_2_ laser for the treatment of AGA was demonstrated in a murine study of shaved mice, by Bae et al.^22^ in 2015. In addition to showing a rapid induction of anagen, the CO_2_ laser was found to cause an upregulation of wingless signaling pathway (Wnt)-10b and β-catenin, previously known pathways implicated in hair growth. Another group conducted a human study enrolling 28 males with AGA.^20^ They showed that the fractional CO_2_ laser used in conjunction with topical growth factors was able to increase hair density and shaft diameter. The study was not suited to show efficacy of fractional CO_2_ laser alone on hair growth induction, but rather that combined treatment could boost hair growth, perhaps through enhancing drug delivery.

### Fractional Ablative Erbium:YAG Laser

Similar to the CO_2_ laser, the chromophore of the 2,940 nm erbium (Er):YAG laser is water. In a randomized controlled trial of 88 mice, it was found that both the Er:YAG laser and topical minoxidil induced anagen more rapidly than induction in an untreated control group.^26^ The effect was greatest with the combined use of laser and minoxidil, demonstrating a potentiating effect. In addition, the authors found that levels of Wnt-10b and β-catenin were higher in the laser and combined treatment groups, further supporting findings that lasers promoted hair growth at least partly through the Wnt-10b and β-catenin pathways. Although this study was conducted on mice, the results are strongly supportive for further examination in human subjects.

### Fractional Nonablative Er Glass Laser

The non-ablative Er glass laser (1,550 nm) has a chromophore of water and works to a depth of 0.4–2.0 mm, causing thermal injury without tissue destruction. The benefits of non-ablative lasers over fractional lasers are the reduction of downtime and less hypopigmentation. We have identified 4 original studies that have used the non-ablative Er glass laser for the treatment of alopecia.

In 2011, Kim et al.^25^ published the pooled results of a mouse and human subject study, marking the first use of fractional lasers for hair regrowth. Murine study results showed that the higher energy, densities, and treatment frequencies were more efficacious for anagen induction. Using these results, the authors initiated a human split-scalp study enrolling 20 male Korean patients with AGA.^25^ Measurements of hair density and growth rate were significantly improved in the treatment sides as compared with controls. Hair density began to decline 1 month posttreatment, showing that like medical treatment, the effects of laser treatment are transient. They were able to see an increase in the Wnt-5a and β-catenin signals, further supporting the role of these pathways in LAHR. A study by Wu et al.^23^ on 140 female mice helped to determine appropriate energy settings for safety and hair regrowth. The group determined that settings above a certain density and/or energy caused scar formation and wound contracture, and induction of anagen phase occurred most rapidly at settings just below this threshold.

In the first study examining the use of fractional lasers on female pattern hair loss, Lee et al.^24^ published a prospective trial of 28 pre- and postmenopausal Korean women. The mean baseline hair density and hair shaft diameter improved after treatment, with none showing progression of disease. The Er glass laser in conjunction with topical finasteride and hair growth factors has also been shown to be effective in 4 cases of patients with recalcitrant AGA.^21^ This series is the only reported use of nonablative Er glass laser in conjunction with topical medications, and the only topical 5α-reductase study to be performed in conjunction with LAHR to date.

### Fractional Nonablative Thulium Laser

The nonablative thulium laser produces a wavelength of 1,927 nm with a chromophore of water. The thulium laser has an approximately 10 times greater absorption coefficient for water compared with the 1,550-nm Er glass laser, and therefore can only penetrate to approximately 0.2 mm. Recently, the effects of the thulium laser on AGA have been studied in 2 randomized controlled trials conducted by the same group.^27,28^ Cho et al.^28^ showed that therapy with a fractional thulium laser, intraperifollicular polydeoxyribonucleotide (PDRN) injections, and application of topical vitamin and mineral solution was superior to mesotherapy with vitamin solution and intraperifollicular PDRN injections. Although the trial was not suited to demonstrate the efficacy of laser treatment alone, it showed support for the use of lasers as a delivery tool for topical drug therapies. The second study by the same group enrolled 10 Korean males with AGA and 18 mice.^27^ Biopsies of treated skin from the mouse study showed no scarring of the skin or destruction of HFs at any of the settings used. The thulium laser treatment caused an increase in hair density and shaft diameter over baseline, and combined thulium laser and topical growth factors showed statistically significant increases over the laser alone. The hair density and shaft diameter exhibited a downward trend from 1 to 4 months of follow-up, once again suggesting that the benefits of treatment may be lost once treatment is discontinued.

## DISCUSSION

Until recently, fractional lasers have not been examined as possible treatments for hair regrowth. There is supporting evidence to suggest that fractional laser therapy alone can aid in hair growth in patients with AGA. Fractional ablative CO_2_,^22^ and fractional nonablative Er glass^21,24,25^ and thulium^27,28^ lasers have all been utilized in human trials, but only the non-ablative Er glass and thulium lasers have been shown to be effective in hair growth on their own. The fractional ablative CO_2_ laser has been shown to be effective with the addition of topical therapies. The fractional Er:YAG laser has only been utilized in mouse studies, where it showed success as a hair growth treatment on its own. Mouse studies have helped establish treatment protocols (laser settings, frequency of treatment). Using similar settings, studies in humans have shown efficacy and safety (Table 1). Common side effects of all laser treatments were mild transient pain and pruritus. Studies comparing LAHR with the addition of topical agents with topical agents alone demonstrated a potentiating and often more rapid induction of hair growth in the combined treatment groups.

In the studies reviewed, the number of sessions ranged from 5 to 15, at weekly to 30-day intervals. Although a lengthy and moderately intensive therapy, patients with poor compliance to daily topical medications may see benefit from regular laser therapy sessions. The ideal duration of therapy and interval between sessions remains to be seen. The transient nature of the improvement on hair growth seen in these studies and the cost of frequent laser treatments may be a prohibitive factor for many patients. There may be a benefit to an aggressive initial induction phase followed by a maintenance phase consisting of less frequent interventions. This proposed laser maintenance therapy remains to be examined as a long-term option. In addition, the studies reviewed required that participants had their hair clipped to a short length to allow for penetration of the laser to the scalp, and to allow for accurate measurement of hair density.

Although the mechanisms of hair regrowth are not fully understood, it has been shown that fractional lasers of different types induce Wnt and β-catenin expression, pathways which are implicated in hair growth.^22,25,26^ Increased vascularity and the microenvironment of healing wounds likely play a role through stimulating HF stem cells, causing an increase of anagen induction. In addition, several studies have demonstrated that adjunctive topical treatments potentiate the effects of LAHR.^21,22,27,28^ Although only 1 FDA-approved topical treatment exists currently, there exist countless potential options. One such option is PRP. PRP currently being a hot topic among plastic surgeons and dermatologists, it has been shown to be effective in induction of hair growth in patients with AGA.^29^ The application of PRP topically in conjunction with laser therapy for alopecia has not yet been studied; however, several reports of the concurrent use of microneedling and PRP have shown favorable results.^30–32^ As microneedling causes similar microinjuries as that of fractional lasers, there is considerable potential for combining PRP and fractional laser therapies. Other investigational drugs, no longer constrained by poor transcutaneous absorption, such as finasteride and growth factors may become good options for combined therapy. It is likely that mild to modest benefit will be seen with many different topical agents, creating a need to develop sensible cost-effective treatment protocols.

Human trials have not yet directly compared variations of laser settings, treatment frequencies, and the addition of different topical treatments. All 4 laser types reviewed here have been shown to aid in hair growth; however, no comparison between different laser types has been made in murine or human studies. Additional studies are required to test these variables. Due to the enormous permutations of treatment protocols, high-quality randomized and split-scalp studies can shed some light on these questions without the need for massive numbers of study subjects. In addition, due to differences in male and female pattern hair loss, it is important to ensure findings are substantiated in both sexes.

## CONCLUSIONS

In short, fractional lasers hold promise as a treatment modality for AGA, particularly with the addition of a topical treatment. Side effects are minimal and patients tend to tolerate treatments well. Patient satisfaction with long-term results remains to be seen. Given that there exist innumerable laser settings, varying treatment frequencies, and countless topical options, many. Many variables remain largely untested. The addition of high-quality, well-designed clinical trials are needed to elucidate the optimal treatment options for LAHR in both men and women with AGA.
